# Glutathione for Food and Health Applications with Emphasis on Extraction, Identification, and Quantification Methods: A Review

**DOI:** 10.3390/metabo13040465

**Published:** 2023-03-24

**Authors:** Anfal Alwan Al-Temimi, Aum-El-Bashar Al-Mossawi, Sawsan A. Al-Hilifi, Sameh A. Korma, Tuba Esatbeyoglu, João Miguel Rocha, Vipul Agarwal

**Affiliations:** 1 Department of Food Science, College of Agriculture, University of Basrah, Basrah 61014, Iraqsawsan.hameed@uobasrah.edu.iq (S.A.A.-H.); 2 Department of Food Science, Faculty of Agriculture, Zagazig University, Zagazig 44519, Egypt; 3 School of Food Science and Engineering, South China University of Technology, Guangzhou 510641, China; 4 Department of Food Development and Food Quality, Institute of Food Science and Human Nutrition, Gottfried Wilhelm Leibniz University Hannover, Am Kleinen Felde 30, 30167 Hannover, Germany; esatbeyoglu@lw.uni-hannover.de; 5 Universidade Católica Portuguesa, CBQF—Centro de Biotecnologia e Química Fina—Laboratório Associado, Escola Superior de Biotecnologia, Rua Diogo Botelho 1327, 4169-005 Porto, Portugal; 6 Cluster for Advanced Macromolecular Design (CAMD), School of Chemical Engineering, University of New South Wales, Sydney, NSW 2052, Australia

**Keywords:** glutathione, oxidative stress, bioactive peptides, natural peptide

## Abstract

Glutathione is a naturally occurring compound that plays a crucial role in the cellular response to oxidative stress through its ability to quench free radicals, thus mitigating the risk of potential damage, including cell death. While glutathione is endogenously present in different plants and animal cells, their concentration varies considerably. The alteration in glutathione homeostasis can be used as a potential marker for human diseases. In the case of the depletion of endogenous glutathione, exogenous sources can be used to replenish the pool. To this end, both natural and synthetic glutathione can be used. However, the health benefit of glutathione from natural sources derived from fruits and vegetables is still debated. There is increasingly growing evidence of the potential health benefits of glutathione in different diseases; however, the determination and in situ quantification of endogenously produced glutathione remains a major challenge. For this reason, it has been difficult to understand the bioprocessing of exogenously delivered glutathione in vivo. The development of an in situ technique will also aid in the routine monitoring of glutathione as a biomarker for different oxidative stress-mediated diseases. Furthermore, an understanding of the in vivo bioprocessing of exogenously delivered glutathione will also aid the food industry both towards improving the longevity and profile of food products and the development of glutathione delivery products for long-term societal health benefits. In this review, we surveyed the natural plant-derived sources of glutathione, the identification and quantification of extracted glutathione from these sources, and the role of glutathione in the food industry and its effect on human health.

## 1. Introduction

Environmental factors including pollution, smoke, ultraviolet rays, and diet can cause damage to somatic cells due to the production of high amounts of reactive oxygen species (ROS)-mediated oxidative stress [1]. In normal functioning of the body, ROS is naturally neutralized by endogenous antioxidants, which is highly cell type-dependent [2,3]. However, to neutralize the overproduction of ROS, exogenous antioxidants are required. These exogenous antioxidants can be delivered through natural sources such as specific fruits and vegetables, via widely consumed processed food fortified with antioxidants, or through medication. In the case of processed food, the supplementation of antioxidants (during fortification or otherwise) has additional benefits, including the enhancement of flavor, aroma, color, health benefits to consumers, and prolonged shelf-life by restricting the oxidation-mediated rancidity of food [4,5,6,7,8]. To avoid any potential harmful impact of synthetic antioxidants, there is a push towards using natural antioxidants found in sources such as plants, fish, and livestock. Some of the examples of natural (water and fat soluble) antioxidants include vitamin E, α-tocopherol, phenolic compounds (curcumin, melatonin, minocycline, resveratrol), flavonoids, essential oils, carotenoids, and low-molecular-weight peptides such as glutathione [2,9]. In this review, we will be focusing on glutathione as a natural antioxidant.

Exogenous glutathione can be delivered through the consumption of a range of fruits and vegetables. Glutathione in fruits and vegetables, when consumed, do not enhance innate glutathione levels; instead, it promotes the production of endogenous glutathione in cells, thus enhancing the innate antioxidant response of cells. The direct consumption of natural fruits and vegetables also avoids the need for the extraction of glutathione. However, advancements in nutraceuticals have led to the extraction, isolation, and purification of glutathione from these natural sources. Traditionally, glutathione is isolated from natural plant-based sources using an acid extraction method, which is similar to other natural compounds.

**Figure 1:**
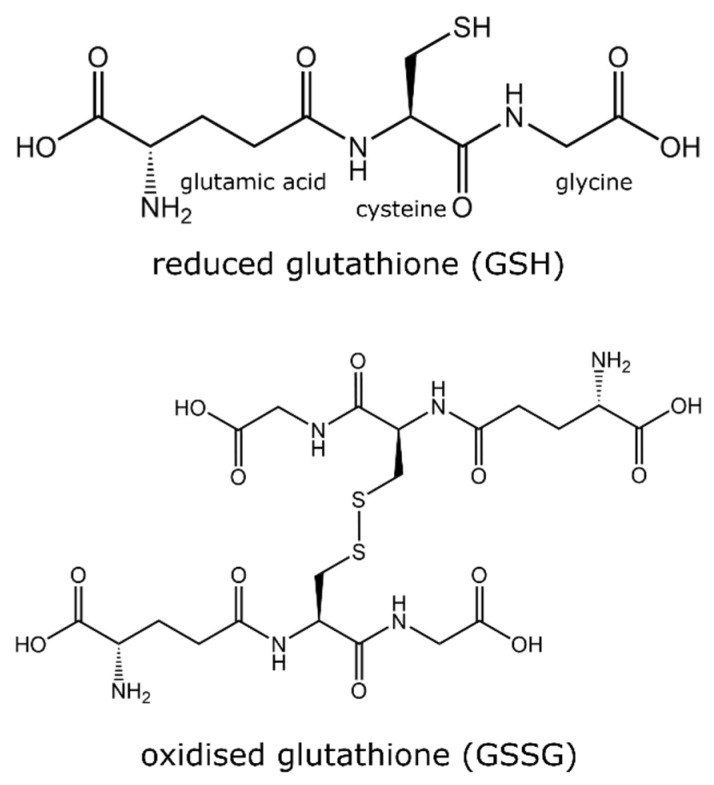
Chemical structure of two isoforms of glutathione (GSH and GSSG).

Glutathione is an essential antioxidant which plays a critical role in human, animal, and plant life. Glutathione is a tripeptide consisting of glutamic–glycine–cysteine and is found in reduced (GSH) and oxidized (GSSG) forms (Figure 1). The ratio between GSH and GSSG is reflective of the level of oxidative stress, where an increase in the ratio (GSSG:GSH) indicates a greater amount of oxidative stress [10]. In normal functioning, GSSG is reduced to GSH by glutathione reductase using a nicotinamide adenine dinucleotide phosphate (NADPH) unit, whereas GSH is oxidized by free radicals (members of the ROS family) to GSSG. In unstressed cells, the amount of GSH is ~90% compared to ~10% GSSG [11]. In neurodegenerative diseases such as Parkinson’s disease and Alzheimer’s disease, the ratio between GSH and GSSG is considerably reduced [10]. The altercation in the ratio of GSH:GSSG is explored as a potential therapeutic marker to diagnose these neurodegenerative diseases. The role of the GSH:GSSG ratio in Parkinson’s disease has been recently reviewed elsewhere [12]. Disturbance in glutathione homeostasis has also been related to the etiology of a range of other diseases including cardiovascular, cancer, diabetes, cystic fibrosis, ageing, and illnesses related to excess oxidative stress [13,14,15,16,17].

Glutathione can also improve the performance of other antioxidants, including vitamin C and E and activate inhibitory enzymes that expose them to a high concentration of oxygen by restoring the disulfide bonds of the enzyme [18]. Glutathione has been proposed to play an important role in preventing iron-mediated programmed cell death in ageing and age-dependent neurodegenerative diseases [19]. Reduction in naturally occurring GSH with ageing increases labile iron, causing ferroptosis (age-related iron-mediated programmed cell death). In this review, we discuss the sources of GSH in plants, recent progress in its identification and quantification, and its importance in the food industry and in human health, with a particular focus on recent studies in the field.

## 2. Plant Sources of Glutathione

**Figure 2:**
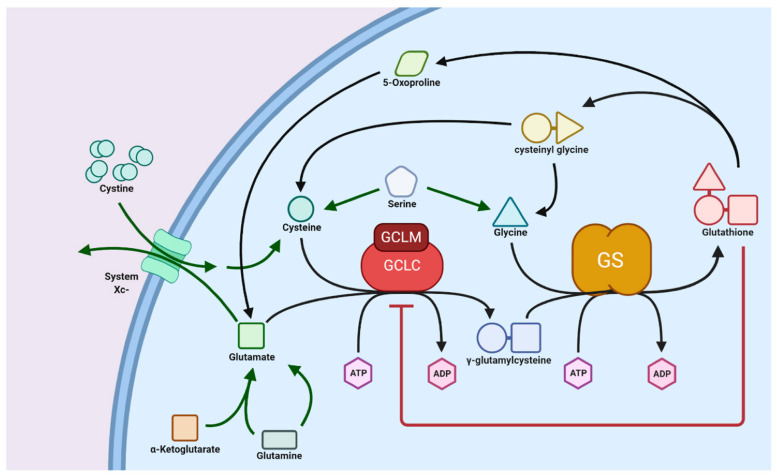
Schematic showing biosynthesis of glutathione. Reproduced with permission from [15].

Glutathione is found in different plant species and its concentration varies in different parts of plants such as leaves, fruit, seeds, fruit peels, petiole, and flowers. For example, fruits such as strawberries, lemons, avocados, and tomatoes are good sources of glutathione with concentrations of ~11.6, 10.5, 15.5, 2.5, and 1.5 mg/100 g, respectively [20]. Some of the examples of vegetables and fruits containing good concentration of GSH includes broccoli, green peppers, oranges, apples, bananas, carrots, spinach, and cauliflower. Being sulfur rich, these vegetables and fruits are known to promote endogenous glutathione production. However, the consumption of plant-based sources may not directly contribute towards glutathione but instead promote its (glutathione) endogenous intracellular biochemical production in cells. Endogenous glutathione in cells boosts functions as a natural antioxidant. For example, the consumption of polyphenols containing fruits and vegetables can promote the intracellular production of glutathione. It has been shown, using a unique transgenic reporter mouse strain, that flavonoids (a type of polyphenol found in fruits and vegetables) promote a simultaneous increase in γ-glutamylcysteine synthetase and intracellular glutathione concentration in muscles [21]. The degradation of glutathione in the gastrointestinal environment during digestion cannot be disregarded [22]. Furthermore, in some tissues, extracellular glutathione needs to be degraded into individual amino acids before transportation inside the cell. This degradation is carried out by the only known glutathionase enzyme, γ-glutamyltranspeptidase, found on the cell plasma membrane. Inside the cell, precursor units are enzymatically linked by combining glutamic acid with cysteine to form γ-glutamylcysteine using the enzyme glutamate–cysteine ligase (GCL). Finally, glycine is added to γ-glutamylcysteine by ATP and Mg^2+^-dependent glutathione synthase (GS) to biosynthesize glutathione (Figure 2) [16,23]. The enzyme GCL can catalyze the entire reaction to synthesize glutathione, but the presence of the GS enzyme can increase the rate of glutathione formation [23].

## 3. Structure and Chemistry of Glutathione

**Figure 3:**
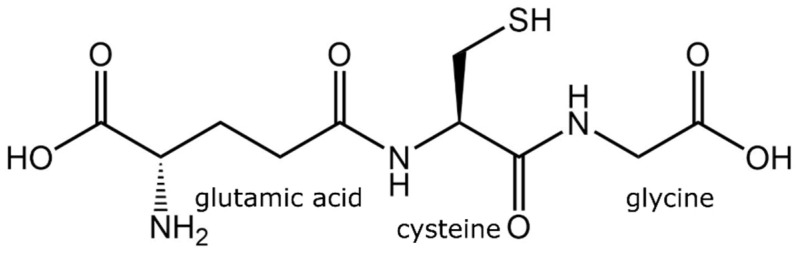
Chemical structure of tripeptides in glutathione.

Glutathione is a low-molecular-weight tripeptide of physiological importance found in living organisms (plants and animals alike). It is found in practically every compartment of plant cells, including the chloroplasts, apoplast, mitochondria, cytosol, vacuole, peroxisomes, and endoplasmic reticulum, and predominantly in mitochondria in animal cells [18,24]. Glutathione has a molecular formula of C_10_H_17_N_3_O_6_S with a molecular weight of 307.32 g/mol and an individual atomic percentage of C = 39.08%, H = 5.58%, N = 13.67%, O = 31.24%, and S = 10.43% [25]. Glutathione was first identified in baker’s yeasts and many animal tissues in 1888 by J. de Rey-Paihade, who named this substance philothion. In 1921, Hopkins suggested that this substance was a dipeptide consisting of cysteine and glutamic acid due to the presence of nitrogen and sulfur atoms, but he did not notice the presence of glycine. In 1927, it was noticed that glutathione was a tripeptide comprising a third amino acid of a low molecular weight, perhaps serine. In 1929, Hopkins conducted the acid hydrolysis of glutathione and demonstrated that it is comprised of a tripeptide containing glutamate, cysteine, and glycine [26]. Then came the research that confirms that glutathione is a tripeptide containing glycine, cysteine, and glutamic acid (Figure 3) [27]. In 1935, Harington and Mead reported a chemical method of glutathione synthesis which paved the foundation for the potential commercial production of glutathione [28]. The commercial production of glutathione commenced in 1950s; however, chemically synthesized glutathione was a racemic mixture of D- and L-isomers, with only L-isomer being physiologically active [29]. Thus, it required a subsequent purification to remove the inactive D-isomer. The global glutathione market was estimated at USD 77 million in 2020 and is projected to reach USD 84 million by 2027 (News Channel Nebraska).

Glutathione comprises an N-terminal glutamic acid (Glu), a central cysteine (Cys), and a C-terminal glycine (Gly). What distinguishes the structure of glutathione is that Glu is linked to Cys in an amide linkage through carboxyl γ and not from carboxyl α position. Gly, the third amino acid, is linked to Cys in an amide linkage, so the glutathione is L-Glu-L-Cys-Gly-γ. The intracellular concentration of glutathione ranges between 0.5 and 10 mM/L depending on the cell type [15,30]. Glutathione contains a large percentage of the Cys, so the thiol group (of Cys) is the leading chemical group regulating its biological and biological functions [31].

The glutathione synthesis pathway is called the γ-glutamyl cycle and has been described in detail previously [13,23,32]. The de novo synthesis of glutathione requires the consecutive action of two enzymes, γ-glutamylcysteine synthetase (γ-GCS) and GSH synthetase [13,33]. The expression of γ-GCS is highly cell type-dependent, which regulates the amount of glutathione formation. The rate-limiting component in the biosynthesis of glutathione is the availability of cysteine. The cellular uptake of cysteine can be enhanced by oxidants such as hydrogen peroxide and electrophilic compounds, which also promote the expression of γ-GCS. However, the phosphorylation of γ-GCS inhibits glutathione synthesis; thus, in one way, glutathione regulates γ-GCS activity via a negative feedback mechanism [13].

## 4. Extraction and Sample Preparation Methods

There are several ways to produce biological peptides including (i) the use of specific enzymes or microorganisms, (ii) acid or basic hydrolysis of the proteins, and (iii) synthetic solid-state peptide synthesis. Explorations of glutathione synthesis were inspired by the observation by Bloch, who studied the biosynthesis of glutathione from its constituent amino acids in rat liver slices and its consequent biosynthetic pathway [34]. The initial research in the field was focused on the exploration of the enzymatic and fermentation production methods of glutathione synthesis, which have been reviewed in detail by Li et al. [29].

Later, glutathione extraction was explored in plant tissues where it was isolated from different parts such as roots, stems, leaves, fruits, and seeds. Table 1 describes the list of different sources of glutathione, extraction methods, type and amount of glutathione, and detection methods. Lata et al. [35]. extracted glutathione from the peels, pulp, and seeds of four varieties of frozen-stored apples. In their work, different parts of the fruit were snap frozen in liquid nitrogen and grounded to obtain a fine powder. Glutathione was extracted from frozen apple powder by homogenizing it in 0.1 M of hydrochloric acid (HCl) containing polyvinylpyrrolidone (PVP), followed by centrifugation at 14,000 rpm for 20 min at 4 °C. The obtained supernatant was reduced with dithiothreitol (DTT) derivatized using monobromobimane. The total glutathione content was determined using fluorometry at 480 nm by exciting the sample at 380 nm [35]. They concluded that the concentration of glutathione in apple seeds was higher than in the core and peel. Xu et al. [36] extracted glutathione from fat-removed and crushed corn embryos by using ultrasound to breakdown cell walls, followed by extraction at 90 °C for 20 min. The total glutathione content in an extracted sample was determined using UV/visible spectroscopy and selecting the maximum absorption wavelength between 190 nm and 600 nm. In their work, they also determined the functional activity of extracted glutathione against neutralizing hydroxyl radical, superoxide anion radical, and DPPH radical. Recently, dry yeast has been used to extract glutathione as a cost-effective, easy to transport alternative to fermentation broths [37]. In this method, hot water (~78 °C) was used to disrupt the yeast cells, releasing intracellular glutathione along with other water-soluble components, following which the suspension was rapidly cooled using ice and centrifuged at room temperature for 20 min. The total glutathione (reduced form—GSH) content was determined from the supernatant using the standard 5,5′-dithiobis-(2-nitrobenzoic acid (DTNB assay) (determining the thiol/sulfhydryl concentration). The extracted GSH was purified and concentrated in a multistep process using ultrafiltration (UF) and nanofiltration (NF) membranes [37]. Baysar and Karatas [38] extracted both reduced and oxidized forms of glutathione (GSH and GSSG, respectively) from fresh apricot fruits from six different species. GSH and GSSG were extracted using perchloric acid (HClO_4_) solution from the homogenized fruits. These homogenized solutions were centrifuged at 4 °C and filtered to remove the precipitated protein. The total glutathione content was determined using high-pressure liquid chromatography (HPLC) [38]. A similar approach using HClO_4_ and HPLC was used to extract GSH and GSSG from eight different species of edible mushrooms [39]. GSH and GSSG were extracted from homogenized mushrooms using the HClO_4_ solution method as outlined in the work by Baysar and Karatas [38]. However, the key difference between the two HPLC methods was the use of different columns and mobile phases [38,39].

The studies outlined above highlight the methods of extracting intracellular glutathione; however, the commercial production from these approaches is not sufficiently cost-effective. To this end, microorganisms with the ability to produce extracellular glutathione have been explored [29]. Examples of such microorganisms include *C. tropicalis* PK233, *Proteus mirabilis* IFO 3849, *S. cerevisiae*, and *C. utilis* 02-08 [40,41]. In the case of *C. utilis* 02-08, the pH of the culture was shown to govern the concentration of extracted glutathione, with a shift in pH to a lower value inducing a significant improvement in the glutathione yield [29]. Furthermore, considering cysteine availability is the rate-limiting factor in glutathione biosynthesis. Perhaps, biochemical and genetic engineering approaches towards increasing the bioavailability of cysteine can profoundly increase glutathione synthesis in food-grade microorganisms, leading to a cost-effective pathway for the commercial production of glutathione.

**Table 1:** Detailing different sources of glutathione, extraction methods, type, and amount of glutathione and detection methods.

Source Material	Extraction Method	Glutathione Amount	Detection Method	Reference
*Beta vulgaris* (leaf), *Prunus persica* (leaf), *Medicago truncatula* (nodule), *Hordeum vulgare* (leaf), *Lycopersicon esculentum* (leaf), *Beta vulgaris* (root), *Trifolium sp.* (leaf), *Oryza sativa* (leaf)	Plant tissue (100–500 mg) was frozen in liquid nitrogen, grounded, 200–1000 μL of cold (4 °C) extraction solution (5% (*w*/*v*) MPA and 1 mM EDTA in 0.1% formic acid), supplemented with 1% (*m*/*v*) polyvinylpolypyrrolidone (PVPP). Homogenates were centrifuged and the pellet was extracted again. The supernatants were combined and syringe-filtered to obtain extracted glutathione.	*Beta vulgaris* (leaf)—152 nmol/g GSH; 23 nmol/g GSSG, *Prunus persica* (leaf)—155 nmol/g GSH; 6 nmol/g, *Medicago truncatula* (nodule)—202 nmol/g GSH; 7 nmol/g, *Hordeum vulgare* (leaf)—not detected GSH; not detected, *Lycopersicon esculentum* (leaf)—707 nmol/g GSH; 47 nmol/g, *Beta vulgaris* (root)—92 nmol/g GSH; 46 nmol/g, *Trifolium sp.* (leaf)—42 nmol/g GSH, *Oryza sativa* (leaf)—252 nmol/g GSH; 13 nmol/g	High-pressure liquid chromatography (HPLC)	[42]
Kappaphycus alvarezii seaweed extract sprayed maize	Leaf tissue (1 g) was frozen in liquid nitrogen, homogenized with 25% H_3_PO_3_ (1 mL) and 3 mL of 0.1 M sodium phosphate–EDTA buffer (pH 8.0). The solution was centrifuged, and the supernatant was collected.	GSH—44–194 µg/g FW	O-phthalaldehyde (OPT)/spectrofluorimetry	[43]
Salvia species (*Salvia nemorosa* L.*, and Salvia reuterana Boiss*)	Leaf tissue (0.2 g) was homogenized with 6% metaphosphoric acid (2 mL) containing 1 mM EDTA, centrifuged, and the supernatant was collected.	GSH–36,352 nmol/g FW; GSSG–86 nmol/g FW	DTNB/UV vis spectroscopy	[44]
Cashew plants (*Anacardium occidentale* L.)	The leaf samples (0.1 g FW) were homogenized in cold 6% trichloroacetic acid (TCA) (*w*/*v*), the homogenate was centrifuged, and the supernatant was collected.	GSH—1.4–2.3 µmol/g	DTNB/UV vis spectroscopy	[45]
Seed of wheat *(Triticum aestivum* L.)	-	2 µmol/g DW	DTNB/UV vis spectroscopy	[46]
*Brassica juncea*	Fresh shoot sample (200 g) was homogenized with 5% *w*/*v* sulfosalicylic acid, centrifuged, and the supernatant was collected.	glutathione 230–350 nmol/min.mg protein	DTNB/UV vis spectroscopy	[47]
*Arabidopsis thaliana*	Leaf or root tissues (500 mg) were homogenized with 1 mL of 5% trichloroacetic acid (TCA), centrifuged, and the supernatant was collected.	GSH—1100–6500 nmol/g FW, GSSG—100–680 nmol/g FW	DTNB/UV vis spectroscopy	[48]
Tomato (*Solanum lycopersicum* L. cv. Condine Red)	Leaf tissue (0.2 g) was homogenized with 2% metaphosphoric acid (2 mL), centrifuged, and the supernatant was neutralized with 0.2 M NaOH before analysis.	GSH+GSSG–350–420 nmol/g FW	DTNB/UV vis spectroscopy	[49]
*Vicia faba* L.	Leaf tissue (0.25 g) was homogenized with 2% metaphosphoric acid (2 mL) and 2 mM EDTA, centrifuged, and the supernatant was collected.	GSH—400–1250 nmol/g FW, GSSG—55–90 nmol/g FW	DTNB/UV vis spectroscopy	[50]
*Cassia alata*	-	GSH—3–7 µmol/g FW	DTNB/UV vis spectroscopy	[51]
Maize (*Zea mays*)	-	Leaves GSH—0.8–1.5 µmol/g FW, roots GSH—0.6–1.1 µmol/g FW	DTNB/UV vis spectroscopy	[52]
Oilseed rape (*Brassica napus* L.) roots	-	GSH—1–1.7 µmol/g FW, GSSG—1.2–2 µmol/g FW	DTNB/UV vis spectroscopy	[53]
Tomato plants (*Solanum lycopersicum* L. cv. Badun)	-	GSH—120–500 nmol/g FW, GSSG—160–430 nmol/g FW	DTNB/UV vis spectroscopy	[54]
Olive fruits (*Olea europaea* L.)	Olive powder (0.4 g) was homogenized with 0.1 M of cold HCl (7 mL), centrifuged, and the supernatant was collected.	GSH—1.3–5.2 mg/g FW, GSSG—0.3–0.7 mg/g FW	Liquid chromatography–electrospray/mass spectrometry (LC–ES/MS)	[55]
Cucumber (*Cucumis sativus* L.) seeds	Plant tissue was homogenized with 5% metaphosphoric acid (5 mL), centrifuged, and the supernatant was collected.	Shoot GSH—580–800 µmol/g FW, GSSG—80–90 µmol/g FW; roots GSH—270–370 µmol/g FW, GSSG—40–58 µmol/g FW	DTNB/UV vis spectroscopy	[56]
Tomato (*Solanum lycopersicum* L. cv. Micro-Tom)	Leaves were homogenized with 3% trichloroacetic acid containing 0.5 mM EDTA, centrifuged, and the supernatant was collected.	GSH/GSSG ratio-4–9	DTNB/UV vis spectroscopy	[57].
Red beetroot vacuoles (*Beta vulgaris* L.)	-	OPT: GSH—0.059 µmol/mg protein; GSSG—0.019 µmol/mg protein, DTNB: GSH—0.091 µmol/mg protein; GSSG—0.031 µmol/mg protein, HPLC: GSH—0.039 µmol/mg protein; GSSG—0.007 µmol/mg protein	OPT/spectrofluorimetry, DTNB/UV vis spectroscopy, HPLC	[58]
Pepper (*Capsicum annuum* L.)	Pericarps and placentas were frozen in liquid nitrogen and crushed into a powder. Powdered tissue (0.4 g) was homogenized with 0.1 M HCl (1 mL), centrifuged, and filtered.	GSH—50–80 µg/g FW, GSSG–2.5–13 µg/g FW	LC–ES/MS	[59]
Raspberry fruit (*Rubus idaeus* L.)	Frozen raspberry tissue was frozen in liquid nitrogen and crushed into a powder. Powdered tissue (5 g) was homogenized with chilled 50 mM sodium phosphate buffer (pH 8.0) containing 5 mM EDTA, centrifuged, and the supernatant was collected.	GSH—40–75 mg/kg DW	OPT/spectrofluorimetry	[60]
Sweet pepper (*Capsicum annuum* L.)	Frozen leaf tissues were homogenized with cold 5% sulfosalicylic acid (10 mL), centrifuged, and the supernatant was collected.	GSH—11–14 mg/100 g FW, GSSG—1.5–2.6 mg/100 g FW		[61]
*Perilla frutescens*	Tissues were frozen in liquid nitrogen and crushed into a powder. Powdered tissue (0.2 g) was homogenized with 0.1% trifluoroacetic acid, centrifuged, and the supernatant was collected.	GSH—3 µg/mL	HPLC	[62]
*Brassica rapa* L.	Frozen powdered sample (0.1 g) was homogenized with 0.1 M of cold HCl (1 mL), centrifuged, and the supernatant was collected.	GSH—200–850 nmol/g FW, GSSG—20–50 nmol/g FW	HPLC	[63]
Upland Cotton (*Gossypium hirsutum* L.)	Plant samples (0.4 g) were homogenized in trichloroacetic acid (4 mL, 5% *v*/*v*), the homogenate was centrifuged, and the supernatant was collected.	GSH—0.05–0.6 µM/g FW	DTNB/UV vis spectroscopy	[64]
Apricot fruits	-	GSH—86–914 µg/g FW, GSSG—17–35 µg/g FW	HPLC	[38]
*Tylophora pauciflora*	-	GSH—61 µg/mg FW	DTNB/UV vis spectroscopy	[65]

## 5. Identification and Quantification of Glutathione

The extraction of glutathione, as outlined in the previous section, requires a purification step after the digestion of the source material (e.g., plant products and microorganisms). The primary contaminants that require removal from extracts are proteins, which are typically precipitated out before quantifying the glutathione content. The quantification of extracted glutathione and its reduced (GSH) or oxidized (GSSG) form have been conducted using a range of analytical techniques, including UV/visible spectroscopy, fluorometry, HPLC, capillary electrophoresis, gas chromatography, mass spectroscopy, nuclear magnetic resonance spectroscopy, and electrochemistry [10,15,17,66,67,68,69,70,71,72,73]. In UV/visible spectroscopy, which is one of the most widely used methods, extracted glutathione is indirectly quantified by determining the amount of thiol groups present in cysteine. This is done using different standard assays such as the DTNB assay, also known as Ellman’s reagent (5,5′-dithiobis (2-nitrobenzoic acid)). In the DTNB assay, the thiol groups in glutathione (or any peptide or protein containing thiol groups) react with Ellman’s reagent, cleaving the disulfide bond to provide 2-nitro-5-thiobenzoate (TNB^−^) [74,75]. TNB^−^ undergoes a further ionization in neutral and alkaline pH in water to form the yellow-colored TNB^2−^ species, the absorbance of which is quantified using a plate reader at 412 nm. The reported limit of detection of the DTNB assay is 0.103 nM [75]. It should be noted that the approach measures all the DTNB reactive thiols contained in the extract and is not selective to glutathione or its forms. However, DTNB does not react with GSSG (oxidized form of glutathione). Recently, a colorimetric method was developed to quantify glutathione in solution using a nanohybrid composed of manganese dioxide and carbon dots [76]. Carbon dots were used to stabilize manganese dioxide in water, whereas manganese dioxide was used to reduce DTMB to form a blue product (the absorbance of which was determined at 655 nm). This blue product faded away in the presence of glutathione, leading to its (glutathione) quantification. This approach exhibited a limit of detection of 0.095 µm with a great selectivity in fetal calf serum [76].

The total concentration of glutathione and its two forms in plants depends on several factors, including the type of plant, the part of plant used in the extraction, the specific extraction method, and the type of test used [77]. For example, Pradedova et al. [58] compared different analytical methods to quantify total glutathione, GSH, and GSSG amounts in red beetroot vacuoles (*Beta vulgaris* L.). They used spectrofluorimetric method with orthophthalic aldehyde (OPT), UV/visible spectroscopy using DTNB, and HPLC. They observed significant differences in the amount of measured GSH, GSSG, and total glutathione depending on the analytical method used. Out of all three methods, DTNB yielded the highest values of all three species: GSH with the concentration of 0.091 µg/mg of dry protein, GSSG with a concentration of 0.031 µg/mg of dry protein, and a total glutathione amount of 0.153 µg/mg of dry protein. They obtained least values in HPLC analysis with a concentration of 0.039 µg/mg, GSSG with a concentration of 0.007 µg/mg, and a total glutathione amount of 0.053 µg/mg of dry protein [58]. Considering the sensitivity of the three techniques, HPLC is regarded as the most sensitive. Therefore, the values obtained by HPLC would be considered the most accurate, particularly considering that UV/Vis spectroscopic analysis can be highly dependent on the sample concentration, leading to an overestimation when using highly concentrated samples during measurements. Furthermore, the use of HPLC also avoids interference from staining reagents such as DTNB and OPT. In addition, both of these methods (DTNB and OPT) are highly dependent on the sample conditions, making them relatively less accurate than the HPLC method. However, HPLC analysis can be a costly endeavor, which inspired the development of electrochemical methods.

The attraction towards electrochemical methods has been based on the fact that it allows for the direct analysis of untreated GSH (GSH can undergo a rapid oxidation to form GSSG). However, electrochemical methods are marred with major obstacles, including the complex composition of the extracted mixtures, leading to electrode fouling [78]. Furthermore, interference from electroactive species comprising ascorbate and cysteine can also lead to unreliable results when analyzing untreated GSH due to interference in redox signals. Interference from solution conductivity also remains a factor which needs to be taken into consideration.

Recently, the electrochemical approach, utilizing the solid-state electrochemistry of cuprous chloride (CuCl), was developed for the detection and quantification of glutathione [79]. In this approach, Au@Cu-MOF (metal–organic framework) nanocapsule was synthesized with Cu-MOF shell and encapsulated Au particles. Using solid-state Au@Cu-MOF nanocapsule-modified electrodes, they obtained a detection limit of 2.5 pM, which was one of the lowest values ever reported. This approach takes advantage of the strong interaction of glutathione towards metal ions (Cu in this case), leading to the formation of a Cu-GSH complex, avoiding electrode fouling by chloride ions (Cl^−^) present in the solution, which in turn caused a sharp reduction in the peak current of CuCl. This change in current was used to quantify the amount of GSH [79]. The strong affinity of glutathione towards metal ions was recently exploited towards its quantification using a handheld multifunctional smartphone platform integrated with a 3D printing portable device as an on-site detection platform technology [80]. The detection approach used in this study was based on the fluorescence resonance energy transfer (FRET) method using silver ions (Ag^+^) and yellow color light-emitting 2,3-diaminophenazine (OxOPD) as the redox components. In this method, Ag^+^ ions oxidize o-phenylenediamine (OPD) to produce OxOPD, causing a change in the FRET signal between 562 and 442 nm. Glutathione preferentially reacts with Ag^+^ ions (due to its high metal ion affinity) and inhibits the production of OxOPD, resulting in a change in FRET peaks; this change in signal can then be used to quantify the glutathione concentration. The limit of detection of this FRET-based method was 0.07 Μm [80], which is significantly lower than electrochemical approaches. The electrochemical analysis of glutathione and its two forms (GSH and GSSG) has been critically reviewed previously [78], while the other analytical methods described in this section have been comprehensively reviewed elsewhere [81], and readers are directed to these reviews for a detailed background.

Taken together from these studies, the precise concentration of the two forms of glutathione can be determined in isolation (one form at a time) using a range of methods, as demonstrated in this section. However, the simultaneous and selective determination of both forms (GSH and GSSG) was a great challenge. To this end, Tsiasioti and Tzanavaras developed a zone fluidics approach utilizing OPT-based fluorometric detection to simultaneously and selectively quantify GSH and GSSG in an automated method [82]. By simple adjustment in solution pH from mild to highly basic, they managed to selectively quantify GSH and GSSG by using fluorometric detection at 340/425 nm. In this method, when determining GSSG, GSH was first masked using N-ethyl-maleimide. The limit of detection of this approach was 60 nmol L^−1^ for GSH and 53 nmol L^−1^ for GSSG, with over 96% reproducibility when performed repeatedly over multiple days. One of the major advantages of this method was the high selectivity of the two forms, with no interference from cysteine present in solution [82]. Based on this study, it can be established that the limit of detection of the zone fluidic method is significantly better than DTNB methods [76]. Despite the superior performance of this fluidics approach, it is not a straightforward method in terms of the development of the experimental setup, which could limit its wide applicability and commercial uptake.

All the methods discussed above are based on extracted samples and in vitro analyses. However, the estimation of both GSH and GSSG is challenging in vivo. For the in vivo detection of GSH and GSSG, techniques such as proton magnetic resonance spectroscopy, mass spectroscopy, electron paramagnetic resonance imaging, fluorescence imaging, and implanted biosensors have been explored [15]. The use of proton magnetic resonance spectroscopy and mass spectroscopy techniques focused on GSH detection in tumors, particularly as an indicator of treatment efficacy and success, taking advantage of the high flux of GSH in and between tumor cells and their microenvironment. However, the exploration of these techniques in models other than cancer still remains in its infancy. The significant cost of these sophisticated instruments and the need for highly skilled operators can be justified for diagnostic purposes; however, their utilization in the commercial production of glutathione in the nutraceutical industry cannot be justified.

## 6. Role of Glutathione in Food

Glutathione plays a pivotal role in physiological processes, including in maintaining redox balance, reducing oxidative stress, removing toxins, and regulating immune system functions. The state and concentration of glutathione in the body is considered a vital sign and a therapeutic target for many chronic and age-related diseases. It has been postulated that glutathione levels can be improved in humans by using fruits and vegetables containing glutathione or amino acids that help its (glutathione) synthesis [9]. Eating green foods, including asparagus, avocado, cucumber, green beans, and spinach raw or slightly steamed, is preferable to preserve both forms of glutathione (GSH and GSSG). Studies have shown that dairy products and cereals are low in glutathione, while fruits and vegetables contain moderate to relatively high amounts of glutathione [9,83]. However, processing, preservation, and cooking methods can alter the glutathione content in food products [83]. In addition to health benefits, glutathione is also responsible for the odor and taste of processed food (e.g., GSH induce a sulfurous odor in processed food) [84,85].

The role of glutathione and its two forms in processed food continues to gain considerable attention, primarily in the development of a better understanding and improve the quality of developed products [86,87]. For example, both GSH and GSSG have been shown to improve the gas-retaining properties of rice batter used in gluten-free bread manufacturing along with the retention of viscoelastic properties (in the obtained dough). Sensory tests revealed that GSSG bread has a significantly less sulfurous odor compared to GSH bread (which has been attributed to the presence of hydrogen sulfide and methyl mercaptan in the GSH bread headspace) [85,88]. The similar effect of GSH on elasticity was also reported on wheat dough. Verheyen et al. [89] used GSH as a replacement to rising yeast (*Saccharomyces cerevisiae*) in wheat dough. They observed a significant softening of the dough after 3 h of fermentation when using a yeast-equivalent amount of GSH instead of active yeast, thus leading the way of potentially replacing dry yeast in bread making. The impact of glutathione in the fermentation food industry is widespread. For example, glutathione supplementation has been shown to impact the stress protection and growth promotion of several lactic acid bacteria species (widely used in the modern fermented food industry and probiotic-based therapeutics) [86]. Supplemented glutathione prevents against all kinds of stress factors, including oxidative, acid, cold, and osmotic stress [86]. Furthermore, the role of glutathione in wine maturation and quality (in terms of a change in odor and taste during storage) have been well documented, regardless of the grape and wine variety [90,91,92]. Taken together, these studies highlight that the supplementation of GSH can bring a dramatic change in the final properties of the processed food and thus play a crucial role in the global food industry. Based on these reports, food can be used as a delivery medium to dose humans with glutathione by using processed food (fortified or supplemented with glutathione). This is particularly useful considering the crucial role glutathione plays in human health and a wide range of diseases.

## 7. Role of Glutathione in Human Diseases

Glutathione plays a pivotal role in physiological processes, including the maintenance of redox balance, neutralizing oxidative stress by promoting the metabolic detoxification of both xenobiotic and endogenous compounds, and regulating the function of the immune system [14]. The depletion of glutathione leads to the (i) release of inflammatory cytokines, (ii) the formation of free radicals, and (iii) the inhibition of some cell functions, all of which have been known to cause many chronic degenerative diseases and functional loss with ageing. Further, glutathione metabolism dysregulation has been shown to induce diseases to the central nervous system, frailty and sarcopenia, infections, chronic liver diseases, metabolic diseases, pulmonary, and cardiovascular diseases [14]. Glutathione contributes to the detoxification of living organisms (by neutralizing ROS), regulates cell proliferation, and is involved in immune function. Knowing the concentration of glutathione makes it possible to detect early diseases because of its significant role in regulating cellular stress [93]. Recent studies have shown that glutathione not only affects normal immune function but also participates in complex immune reactions such as fever. The complex role of glutathione was discovered in patients who do not develop a fever during infection [94]. Generally, fever is associated with oxidative stress; therefore, it was believed that the antioxidant properties of glutathione can reduce its incidence. Studies have shown that even a low glutathione level is sufficient to reduce fever. However, the main problem arises when the primary symptoms of infection (i.e., fever) is not manifested. Therefore, it can be proposed that regardless of fever, patients with infection should be treated with glutathione. The impact of glutathione on fever has been comprehensively reviewed in a recent review by Wrotek et al. [94].

The spread of chronic diseases and premature aging at present has led to increased studies in the field of antioxidants, especially glutathione, because of its importance in reducing these diseases. Nutrients, including antioxidants and glutathione, have been recommended to be taken for a very long time or for a lifetime to have an apparent effect on humans. The high cost of these nutrients remains an inhibitory factor limiting their uptake. However, the uptake of exogeneous glutathione consumed as a supplement is still debated [9]. In the meantime, attempts are being made to increase the amount of glutathione in food, such as animal tissues (meat) through the application of modern genetic and reproductive techniques, and improve the levels of these antigens in animals’ bodies. Thus, it is possible to improve the quality of meat products, extend their storage life, and obtain high-quality meat which contains good proportions of glutathione and other antioxidants important to human consumers [95]. Becker et al. [96] conducted a pilot clinical study on the effect of thiol-containing antioxidants (glutathione, α-lipoic acid, and N-acetylcysteine) on the recovery and survival of malnutrition syndrome kwashiorkor children. Kwashiorkor disease is a severe form of malnutrition reported to be associated with oxidative stress. In their study [96], children suffering from the disease were randomly assigned to either a standard treatment (recommended by the WHO) or one of the three study groups receiving either 2 × 600 mg of reduced glutathione (GSH) or 2 × 50 mg of α-lipoic acid or 2 × 100 mg of N-acetylcysteine per day. In a 20-day follow-up, they observed that GSH and α-lipoic acid supplementation had a strong correlation with patient survival rate, as determined from initial skin lesions, blood glutathione levels, glutathione, and total protein concentrations. This study outlined the therapeutic potential of glutathione supplementation in reducing the incidence of severe acute malnutrition caused by oxidative stress [96]. Manley [97] stated that the diet of people plays a vital role in determining the levels of glutathione inside the body, as it was noted that people whose diet depends on red and white meat have higher levels of glutathione, up to 2.3 μmol/kg compared to 1.9 μmol/kg in vegetarian people. The reason behind this disparity is the low amount of vitamin B12 in meat. When consumed, vitamin B12 from meat regulates the sulfur biochemical pathway to produce glutathione.

Glutathione also plays a critical role in infections of the pulmonary system. Studies have shown that glutathione depletion increases a person’s susceptibility towards infections such as tuberculosis. The depletion of glutathione in peripheral blood mononuclear cells and red blood cells has been observed in tuberculosis patients compared to a healthy control [98]. To mitigate this shortage, patients were supplemented with liposomal glutathione. The supplemented liposomal glutathione significantly enhanced the T-cell response in HIV-positive patients with tuberculosis infection, while simultaneously reducing the level of free radicals and immunosuppressive cytokines (interleukin-10 (IL-10) and the transforming growth factor-β (TGF-β)) relative to the placebo-controlled group [99]. The observed infection control has been reasoned to the anti-mycobacterial effects of glutathione [93]. Glutathione also regulates natural killer (NK) cell activity in innate intracellular bacterial infections in tuberculosis-infected patients. In tuberculosis, the cytolytic activity of NK cells is critically impaired in patients with low glutathione levels. The treatment of such patients with N-acetylcysteine can recover and regain the cytolytic activity of NK cells and their efficiency in tackling tuberculosis infection [93,100,101]. Glutathione also regulates dendritic cell maturation and their function in the differentiation of native T-cells. An increase in glutathione has been shown to upregulate IL-12 production by dendritic cells, where (IL-12) is responsible for T-cells differentiation, with a significant potential in the infection control [93]. Due to the fundamental role of glutathione in multiple immune cell types and their functions, it (glutathione) has been shown to be critical in different infections, including HIV. The role of glutathione in HIV has been reviewed in detail elsewhere [93].

Recently, Polonikov [102] hypothesized the role of glutathione in the severity of COVID-19. Using the clinical data of four patients, they observed an inverse correlation of the amount of glutathione and COVID-19 severity in patients where severe cases had significantly low endogenous glutathione levels, higher ROS, and a higher ROS/glutathione ratio in plasma than patients with mild disease [102]. It was hypothesized that glutathione deficiency can increase the susceptibility for the uncontrolled replication of SARS-CoV-2 (COVID-19) virus, leading to a significant increase in viral loading regardless of other factors such as aging, chronic disease comorbidity, and smoking. In this study, it was proposed that the long-term oral administration or parenteral injection of N-acetylcysteine (a precursor of endogenous glutathione synthesis) could be used as an efficient therapy for COVID-19 patients with a serious illness [102,103,104]. The primary reasons behind the use of N-acetylcysteine over pure glutathione as a therapeutic strategy include the low bioavailability and short half-life (2 min) of glutathione when administered orally or intravenously [16,105]. The reason for its low bioavailability is the rapid degradation of glutathione by intestinal and hepatic gamma-glutamyl transferase [22]. To mitigate intestinal degradation, the orobuccal or sublingual form of glutathione delivery has been developed, which was shown to increase the level of GSH and the GSH/GSSG ratio [22,106].

**Figure 4:**
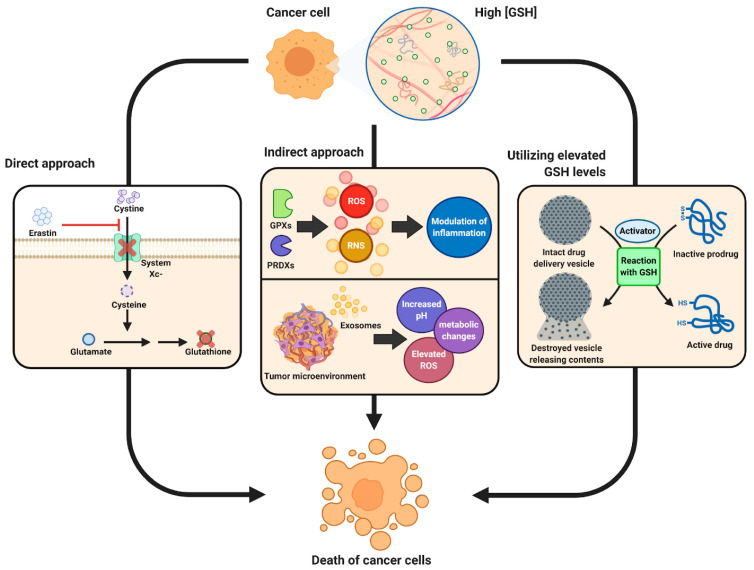
Schematic showing therapeutic role of glutathione in cancer tumor biology. Reproduced with permission from [15].

While most work has implicated glutathione in a restorative and preventative role in the cellular function and pathologies of difference diseases, a fine balance must be maintained in glutathione homeostasis as glutathione has shown to have both protective and pathogenic roles. For example, changes in the glutathione antioxidant system and disruption in its homeostasis have been implicated in tumor initiation, progression, and treatment response [15]. At high concentrations in tumor cells, glutathione has been shown to cause tumor progression and an increased resistance to chemotherapeutic drugs. Therefore, focus has been drawn towards targeting the glutathione antioxidant system in tumor cells as a therapeutic approach using drugs to target glutathione directly, indirectly, or by using glutathione-based prodrugs (Figure 4) [15]. The concept of a therapeutic approach towards glutathione homeostasis in cancer has been recently reviewed by Kennedy et al. [15], and readers interested in this topic are directed to that comprehensive review.

Glutathione prodrugs or amino acid precursors can also regulate ageing. In ageing, mitochondria start to dysfunction due to the decline in de novo glutathione synthesis, resulting in enhanced oxidative stress, thereby making cells susceptible to microbial infection and death. This compromised mitochondrial function is driven by the combination of a shortfall in glutathione precursor amino acids (cysteine and glycine) and the accumulation of homocysteine (a toxic transsulfuration/glutathione biosynthesis pathway intermediate) [93]. Therefore, it is believed that a supplementation with glutathione precursors can alleviate mitochondrial dysfunction and prevent cell death, restricting ageing. However, the mode of supplementation must be carefully considered, as described previously in this review.

## 8. Conclusions and Future Work

Glutathione continues to draw significant research attention due to its critical role in virtually all animal and plant species. Glutathione plays a central role as an antioxidant against ROS, thus regulating the signaling pathways involving cellular homeostasis. The role of glutathione is also important in different cellular processes including proliferation, apoptosis, immune system modulation, and the cellular component metabolism. The functional outcome of glutathione is regulated by the ratio between its reduced form (GSH) and oxidized form (GSSG), which is indicative of the level of oxidative stress. While endogenous glutathione (with a higher concentration of GSH) can optimally regulate cellular homeostasis, in the cases of upregulated ROS production, exogeneous glutathione is warranted.

While the health benefits of exogenous glutathione are seemingly well-established now, the mode of delivery and question of the use of natural food products, such as fruits and vegetables as (glutathione) sources, remains under dispute. There is a great need to develop experimental strategies to characterize the glutathione metabolism upon consumption (both synthetic and natural) in vivo. Perhaps a systematic in vivo study comparing the exogenous glutathione metabolism compared to endogenous (synthesized using either glutathione precursor amino acids or glutathione-based prodrugs) can provide compelling outcomes to settle this debate. Besides this, the identification and determination of glutathione either as an intact molecule or its two forms require them to be extracted and analyzed. While some in vivo studies have shown promise in the identification and quantification of glutathione forms, these have been limited to a specific field of research particularly focusing on diagnosing a disease type (e.g., cancer). There are perceived gaps in the field, i.e., (i) the development of analytical and imaging techniques to allow for the routine monitoring of glutathione and its forms in healthy adults similar to the periodic screening of different diseases used as a diagnostic tool or a forecasting method to predict future ailments, and (ii) the in vivo determination of glutathione in plants and plant products, which remains greatly unexplored. Furthermore, the potential of food fortification with glutathione as a therapeutic delivery mechanism to the general population could be explored as clinical and greater epidemiological case studies, particularly in light of its (glutathione) therapeutic role in a range of diseases, including infections, cardiovascular, diabetes, and cancer. The fortification of food can have a dual effect of (i) improving food quality, as observed in different industries, including bakery and wine, and (ii) replenishing glutathione resources in humans to vane off future illness caused by oxidative stress and maintain long-term healthy lives.
